# Supplementary material for: Integral, mean and covariance of the simplex-truncated multivariate normal distribution
Source: PLoS One. 2022 Jul 22;17(7):e0272014. doi: 10.1371/journal.pone.0272014 (PMC9307197; doi:10.1371/journal.pone.0272014)
Supplement: S1 Text — (PDF) [file pone.0272014.s001.pdf]

S1 Text: Identifying all ellipse arcs within a linearly truncated domain for the LIN-ESS algorithm

Integral, mean and covariance of the simplex-truncated multivariate normal distribution

Matthew P. Adams[1,2,3,*]

[1] *School of Mathematical Sciences, Queensland University of Technology, Brisbane QLD 4001, Australia.*

[2] *Centre for Data Science, Queensland University of Technology, Brisbane QLD 4001, Australia.*

[3] *School of Chemical Engineering, The University of Queensland, St Lucia QLD 4072, Australia.*

[*] *Email: mp.adams@qut.edu.au*

In the "Materials and methods" section of the manuscript it was stated that the third step of the LIN-ESS algorithm for ST-MNDs is to identify all values of θ on the ellipse defined by Eq (9) that fall within the domain of the ST-MND. Here the mathematical details required to accomplish this third step of the LIN-ESS algorithm are provided, for any domain truncated by linear constraints (of which ST-MNDs are a special case). The mathematical procedure described here is similar to that provided in Gessner *et al.* (reference [13] in the manuscript text), although some different trigonometric relations are utilised, so these relations are described carefully here.

Finding all values of θ within the truncated domain requires finding all values of θ that act as boundaries between arcs of the ellipse falling inside and outside this domain. For example, in the sampling step shown in Fig 1, there are four such boundary values of θ which form end-points for each of the two ellipse arcs that are within the truncated domain (thick red arcs in Fig 1).

Boundary values of θ will typically occur at the intersection of the ellipse and one of the domain constraints.

(It is possible that these boundary values of θ could occur at the intersection between the ellipse and more than one domain constraint simultaneously, but this is highly unlikely and thus can be ignored since the vectors $\boldsymbol{y}_t$ and $\boldsymbol{\nu}$ that generate the ellipse defined by Eq (9) are both drawn from continuous distributions.) It is denoted that θ_i represents values of θ on the ellipse which intersect with the ith linear domain constraint. There will typically be zero or two values of θ_i for each ith constraint, because the ellipse will intersect each linear constraint in either zero or two places. (A single value of θ_i for a constraint indicates a tangential intersection, which is again highly unlikely.) All domain constraints require checking, to identify all values of θ_i.

However, not all intersections θ_i between the ellipse and a domain constraint will result in a boundary value of θ. For example, in Fig 1, both the $x_1 \geq 0$ and $x_2 \geq 0$ constraints possess intersections with the ellipse that do not separate arcs outside and inside the truncated domain (intersection of red ellipse and dashed black lines in Fig 1). Thus, boundary values of θ are a subset of the obtained θ_i. However, since the aim is to identify all values of θ within the truncated domain, it is not necessary to identify which values of θ_i are boundary values. Instead, one can simply examine all ellipse arcs bounded by adjacent values of θ_i to see if these arcs fall within the domain or not. Therefore, the main problem then becomes to find all values of θ_i.

Comparing the above considerations with the inequality given by Eq (6), intersections of the ith constraint with the ellipse (and thus the desired values of θ_i) occur when

$$\boldsymbol{A}_i \boldsymbol{y} + c_i = 0, \tag{S1.1}$$

where here $\boldsymbol{A}_i$ and c_i should be understood as the ith rows of A and $\boldsymbol{c}$ respectively. (The number of rows in both A and $\boldsymbol{c}$ indicates the number of constraints that need to be separately considered for identification of values of θ_i.) From combination of Eq (9) and Eq (S1.1), the problem then becomes the identification of intersection points with a value of θ_i satisfying

$$\boldsymbol{A}_i \left(\boldsymbol{y}_t \cos\theta_i + \boldsymbol{\nu} \sin\theta_i \right) + c_i = 0, \tag{S1.2}$$

for each of the constraints. Since the ellipse will typically intersect each linear constraint in either zero or two places, Eq (S1.2) similarly is expected to have either zero or two solutions for θ_i.

To solve Eq (S1.2) for θ_i, the non-negative distance r_i is introduced,

$$r_i = \sqrt[+]{(\boldsymbol{A}_i \boldsymbol{y}_t)^2 + (\boldsymbol{A}_i \boldsymbol{\nu})^2}. \tag{S1.3}$$

If $|c_i| > r_i$, there are no real solutions for θ_i, which indicates that there are no intersections between the ellipse and the ith constraint. In the unlikely event that $|c_i| = r_i$, there is one real solution for θ_i representing a tangential intersection between the ellipse and the ith constraint.

If $|c_i| < r_i$, there are two real solutions for θ_i, indicating two intersections between the ellipse and the ith constraint. In this latter case, the angle $\alpha_{i,1} \in (-\pi, \pi]$ is introduced which satisfies

$$\cos \alpha_{i,1} = \frac{\boldsymbol{A}_i \boldsymbol{y}_t}{r_i}, \tag{S1.4}$$

$$\sin \alpha_{i,1} = \frac{\boldsymbol{A}_i \boldsymbol{\nu}}{r_i}. \tag{S1.5}$$

Substitution of Eq (S1.4) and Eq (S1.5) into Eq (S1.2), together with usage of an appropriate trigonometric identity, yields

$$\cos\left(\pm\left(\alpha_{i,1} - \theta_i\right)\right) = \frac{-c_i}{r_i}. \tag{S1.6}$$

Finally, the angle $\alpha_{i,2} \in [0, \pi]$ is introduced which satisfies

$$\theta_i = \alpha_{i,1} \pm \alpha_{i,2}, \tag{S1.7}$$

Rearrangement and substitution of Eq (S1.7) into Eq (S1.6) yields

$$\cos \alpha_{i,2} = \frac{-c_i}{r_i}. \tag{S1.8}$$

Thus, finding the two values of θ_i indicated by Eq (S1.7) requires finding the values of angles $\alpha_{i,1}$ and $\alpha_{i,2}$ which satisfy Eq (S1.4), (S1.5) and (S1.8). (As an aside, Eq (S1.7) can also be used when there is a tangential intersection, because in this case $\alpha_{i,2}$ will be equal to either zero or π, thus yielding one value for angle $\theta_i \in [0, 2\pi)$.)

Care must be taken when calculating angles $\alpha_{i,1}$ and $\alpha_{i,2}$ from the inverse trigonometric relations described in Eq (S1.4), (S1.5) and (S1.8). It is recommended that each of these angles are calculated in two steps. First, corresponding acute angles, which are denoted here as $\alpha^*_{i,1}$ and $\alpha^*_{i,2}$, both of which are only defined in

the first quadrant, $\alpha^*_{i,1}, \alpha^*_{i,2} \in \left[0, \frac{\pi}{2}\right]$, can be calculated as

$$\alpha^*_{i,1} = \arctan\left(\left|\frac{\boldsymbol{A}_i\boldsymbol{\nu}}{\boldsymbol{A}_i\boldsymbol{y}_t}\right|\right), \tag{S1.9}$$

$$\alpha^*_{i,2} = \arccos\left(\left|\frac{c_i}{r_i}\right|\right). \tag{S1.10}$$

Second, the values of the required angles $\alpha_{i,1}$ and $\alpha_{i,2}$ are calculated from $\alpha^*_{i,1}$ and $\alpha^*_{i,2}$ via the following formulae:

$$\alpha_{i,1} = \begin{cases} -\pi + \alpha^*_{i,1}, & \text{if } \boldsymbol{A}_i\boldsymbol{y}_t < 0 \text{ and } \boldsymbol{A}_i\boldsymbol{\nu} < 0, \\ -\alpha^*_{i,1}, & \text{if } \boldsymbol{A}_i\boldsymbol{y}_t \geq 0 \text{ and } \boldsymbol{A}_i\boldsymbol{\nu} < 0, \\ +\alpha^*_{i,1}, & \text{if } \boldsymbol{A}_i\boldsymbol{y}_t \geq 0 \text{ and } \boldsymbol{A}_i\boldsymbol{\nu} \geq 0, \\ \pi - \alpha^*_{i,1}, & \text{if } \boldsymbol{A}_i\boldsymbol{y}_t < 0 \text{ and } \boldsymbol{A}_i\boldsymbol{\nu} \geq 0, \end{cases} \tag{S1.11}$$

$$\alpha_{i,2} = \begin{cases} \pi - \alpha^*_{i,2}, & \text{if } c_i > 0, \\ \alpha^*_{i,2}, & \text{if } c_i \leq 0. \end{cases} \tag{S1.12}$$

In summary, real solutions θ_i to Eq (S1.2) are calculated from Eq (S1.3), (S1.7) and (S1.9)-(S1.12), and these solutions exist if and only if $|c_i| \leq r_i$.

Once values of θ_i have been calculated (if they exist) for all constraints, they are organised in a convenient manner so that arcs of the ellipse that they bound can be systematically checked to see if they are within the truncated domain or not. To accomplish this, found values of θ_i that are outside of $0 \leq \theta_i < 2\pi$ are transformed to satisfy this inequality by addition or subtraction of appropriate multiples of 2π. All found angles θ_i are then ordered from smallest to largest in a column vector of angles $\boldsymbol{\theta}_\mathrm{F}$, whose qth element is referred to as $(\theta_\mathrm{F})_q$. Subsequently, an additional angle, equal to the sum of 2π and the smallest θ_i, is appended to the end of this vector, i.e. $\boldsymbol{\theta}_\mathrm{F} \leftarrow [\boldsymbol{\theta}_\mathrm{F} \;\; (\theta_\mathrm{F})_1{+}2\pi]^\top$. This additional angle ensures that all parts of the ellipse are bounded by arcs between consecutive elements within $\boldsymbol{\theta}_\mathrm{F}$. This vector $\boldsymbol{\theta}_\mathrm{F}$ is a convenient organisation of the found values of θ_i for arc checking.

All arcs bounded between the angles that are consecutive elements within $\boldsymbol{\theta}_\mathrm{F}$ are checked to see if the values of θ these arcs contain fall inside or outside the truncated domain. This checking procedure may be undertaken as follows:

1. The total number of arcs to be tested, denoted Q, is one less than the total number of elements in the

vector $\boldsymbol{\theta}_{\mathrm{F}}$. Denote q as the current arc to be tested, and set $q = 1$.

2. Choose any value of θ satisfying $(\theta_{\mathrm{F}})_q < \theta < (\theta_{\mathrm{F}})_{q+1}$, and substitute this value of θ into the ellipse equation, Eq (9), to identify its location $\boldsymbol{y}$.

3. If this value of $\boldsymbol{y}$ satisfies all constraints in the inequality given by Eq (6), then all values of θ that exist within the arc $(\theta_{\mathrm{F}})_q < \theta < (\theta_{\mathrm{F}})_{q+1}$ correspond to locations within the truncated domain. Otherwise, if any of the constraints in Eq (6) are not satisfied, the corresponding range of θ should be disregarded (since these θ correspond to an arc that falls outside the truncated domain).

4. If the current arc q is equal to total number of arcs Q to be tested, then all arcs have now been checked. Otherwise, set $q \leftarrow q + 1$ and go back to Step 2.

This arc checking procedure therefore identifies all values of θ that exist within the truncated domain, which completes the third step of the LIN-ESS algorithm described in the "Materials and methods" section of the manuscript.
